# Toward psychiatry as a ‘human’ science of mind. The case of depressive disorders in *DSM-5*

**DOI:** 10.3389/fpsyg.2014.01517

**Published:** 2015-01-05

**Authors:** Marco Castiglioni, Federico Laudisa

**Affiliations:** Department of Human Sciences “R. Massa”, University of Milano-BicoccaMilan, Italy

**Keywords:** depression, *DSM*, reductionism, normativity, diagnosis, bereavement, constructivism, naturalism

## Abstract

The aim of this paper is to argue that a strictly reductionist approach to psychiatry represents a theoretical and clinical obstacle to a fruitful synthesis between neurobiological and sociocultural aspects of the sciences of mind. We examine the theoretical and practical motivations underlying this approach, by analyzing the case of depressive disorders, as defined in the fifth edition of the *Diagnostic and Statistical Manual of Mental Disorders* (*DSM*), and the related removal of the “bereavement exclusion clause.” We first explore the claim that *DSM* is atheoretical, observing that, far from being atheoretical, *DSM* adopts an implicit, biologically inspired view of the mind; we show that such a view leads to a sort of circularity in the definition of depressive disorders, in which psychopharmacology seems to play a key role. We then turn to further problems deriving from this position, analyzing the issue of placebo effects in the treatment of depressive disorders and the philosophical question of normative preconditions for psychopathological diagnosis. Finally, we address the issue of subjectivity, which, together with the related aspect of the subject’s relational context, appears to be crucial to any scientific theorizing about mental disorders, despite *DSM*’s attempt to exclude it. Our defense of a non-reductionist view of mental disorders, however, does not imply that we endorse any sort of metaphysical dualism, or anti-diagnostic or anti-psychiatric positions. On the contrary, we argue that the adoption of a reductionist position actually undermines the theoretical and clinical accuracy in explaining depressive disorders.

## INTRODUCTION

Over the past two decades, the landscape of psychiatry^1^ has changed dramatically: impressive developments within neuropsychology have fuelled an explosion of interest in the biological bases of mindedness (Andreasen, 1984, 2001; White et al., 2012; Walter, 2013), often leading the subjective, relational, and contextual factors influencing normal and abnormal behavior to be overlooked. Despite claims about the social and discursive dimensions of mind and personhood, particularly those informed by social constructionism (Harré, 1998; Borch-Jacobsen, 2009; Gergen, 2009), biologically inspired views seem to currently prevail, at least in relation to the explanation of mental disorders. In this paper, we examine the theoretical and practical motivations underlying this predominance, by analyzing the case of depressive disorders as depicted in the American Psychiatric Association’s (APA) *Diagnostic and Statistical Manual of Mental Disorders* (*DSM*). In particular, we take the definition of major depressive disorder (MDD) and the related omission of the “bereavement exclusion clause” as instances of a more general view informing the approach brought to bear in *DSM-5* (American Psychiatric Association [APA], 2013a). We claim that *DSM* system implicitly assumes in the case of MDD a reductionist stance and that such a position is likely to represent a serious clinical and theoretical obstacle to developing a “modern synthesis” within the sciences of mind.

As is well known in the philosophy of science, the term *reductionism* may have different meanings according to what exactly is held to be reduced: we may be reductionist in trying to reduce either the *language* of a theory T to the language of a reducing theory T′, or the *laws* of T to the laws of T′, or the basic *entities* described by T to those described by T′ (see the review in van Riel and van Gulick, 2014). Although there is no rigorous, universally accepted theory of reduction, in this paper, we will refer to a “reductionist approach” as an approach that tends in principle to reduce psychological and psychopathological phenomena to their neurobiological correlates.

We attempt to show that despite its allegedly descriptive and atheoretical standpoint, the *DSM*, as currently formulated, implicitly relies on a strongly individualistic view of depression and, more generally, of a range of mental disorders: it supports an unwarranted view of the “mind” and the “human being” as essentially reducible to their neurophysiological bases, at the expense of failing to take into account the relational, social, and cultural factors influencing mental suffering.

The paper is organized as follows. First, we examine the claim that *DSM* is atheoretical. In this regard, we emphasize that, far from being atheoretical, *DSM* adopts an implicit, biologically inspired view of the mind, and we suggest that such a view leads to a sort of circularity in the definition of depressive disorders, in which psychopharmacology seems to play a key role. We also address the problem of reliability vs. validity in the diagnostic process; this issue has been raised by the major critics of the current *DSM* edition as a systematic whole, strictly linked to the importance of taking into due account the subjective and contextual factors for diagnosing depressive disorders. We then turn to further problems deriving from an atheoretical position, discussing the issue of placebo effects in the treatment of depressive disorders and the philosophical question of normative preconditions for psychopathological diagnosis. Finally we return to the issue of subjectivity, which, together with the related issue of the subject’s relational context, appears to be crucial to any scientific theorizing about mental disorders, despite *DSM*’s attempt to exclude it. As we hope to make clear, however, defending a non-reductionist view of mental disorders need not imply endorsement of any kind of metaphysical dualism, or of anti-diagnostic or anti-psychiatric positions. On the contrary, we argue that the adoption of a reductionist position actually undermines the very accuracy in explaining depressive disorders in theoretical and clinical terms.

It must be clearly emphasized that our critical remarks are focused on a specific disorder (MDD). This critical stance need not imply a dismissive attitude toward the whole diagnostic system proposed in the manual: MDD formulation by *DSM-5* is especially wanting from our point of view, but we acknowledge that in several other respects and with reference to other pathologies (i.e., neurocognitive disorders and personality disorders) *DSM-5* may be seen as a significant advancement (Migone, 2013; Maj, 2014).

## DEPRESSIVE DISORDERS FROM THE ATHEORETICAL PERSPECTIVE OF *DSM*

The publication of *DSM*’s fifth edition in 2013 was preceded, and is still accompanied, by strong debate, both within the scientific community and in the media (Wakefield, 2010; Angell, 2011a,b; Spitzer, 2011; Frances, 2013; Insel, 2013). The issue at stake is that *DSM-5* may lead to the increasingly widespread “medicalization” of psychology. It is suggested that – also due to its impact via the social media – *DSM-5* is likely to turn into a true “social representation” (Moscovici et al., 2001) with the power to strongly influence clinical practice, pushing it in the direction of the large-scale prescription of drugs. Notably, among the most distinguished critical voices taking part in this debate, we find Robert Spitzer and Allen Frances, the editors of *DSM-III* (American Psychiatric Association [APA], 1980) and *DSM-IV* (American Psychiatric Association [APA], 1994), respectively.

One of the most significant and controversial changes in the new edition of *DSM* is its dropping of the “bereavement exclusion clause,” that is to say, its omission of the last bulwark still present in *DSM-IV* that had allowed a distinction to be drawn between depression and normal sadness (Horwitz and Wakefield, 2007; Horwitz, 2011; Wakefield and First, 2012; Wakefield and Schmitz, 2012). This clause prevented people who had experienced the loss of a loved one in the two months prior to the onset of symptoms from being diagnosed with MDD. Frances (2013), head of the *DSM-IV* task force, regretfully observes that dropping the bereavement exclusion clause means confusing mourning with melancholia:

“*DSM-5* has made it easier to diagnose MDD among the bereaved, even in the first weeks after their loss. This was a stubbornly misguided decision in the face of universal opposition from clinicians, professional associations and journals, the press and 100s of 1000s of grievers from all around the world” (Frances, 2013, p. 103).

In *DSM-5* bereavement comes to be considered “a severe psychosocial stressor,” which only represents an “additional risk” (American Psychiatric Association [APA], 2013b) for the outset of depressive disorders in people already constitutionally inclined toward depression. The removal from *DSM-5* of the bereavement exclusion clause seems to be the logical outcome of a process initiated many years earlier with the publication of *DSM-III* (American Psychiatric Association [APA], 1980). In this section we try to illustrate some of the assumptions that have produced this outcome. It is not our intention to retrace here the complex history of depression in Western culture and the intricate taxonomic issues that have arisen over time and which are also reflected in the various editions of the *DSM* (for a detailed account see: Ehrenberg, 1998; Shorter, 2009, 2013). We shall only refer to a general and widely accepted distinction concerning the origin of depressive symptoms: the traditional dichotomy between “endogenous” depression (once called *melancholia*), that is to say, biologically based depression arising in the absence of any apparent external reason on the one hand, and reactive depression, that is, a depression triggered by negative external circumstances on the other (Pignarre, 2001). This distinction, which informed the history of psychopathology for centuries and still seems meaningful to the extent that it retains its status as a Western common sense belief, first lost support and ultimately was totally abandoned by contemporary psychiatry. The outcome has been the “triumph of major depression.” “Continuing this tradition seemed relatively straightforward, but instead the tradition derailed and the two depressions became collapsed into one” (Shorter, 2009, p. 158). *DSM-III* appears to have represented a sort of point of no return in the direction of the demise of this traditional distinction.

“[…] When *DSM-III* launched ‘major depression’ in 1980, psychiatrists found themselves quite without defense. Many sensed that there was a big problem in conflating endogenous depression and reactive unhappiness: we were told that breaking up with your boyfriend was on a par with lying curled into fetal, melancholic ball. Both could be major depression as long as the “Chinese menu” of criteria was satisfied” (Shorter, 2013, p. 195).

How did this come about? The main reason was that starting from its third edition, *DSM* has been presented as an *atheoretical* and solely descriptive tool, that is to say, as independent of any particular psychological or psychiatric theory. This decision was justified by Spitzer, who headed up the *DSM-III* task force, on the basis of the reasonable practical need to formulate a common language for the scientific community, which up to that time had been divided into different factions to such an extent that it was virtually impossible to obtain diagnostic agreement on given patients among clinicians from different theoretical schools. Hence the decision to define and categorize the different mental disorders solely on the basis of descriptive symptoms, without attributing them to any (set of) causes, whether internal or external, because this would imply a specific theoretical system. This *DSM* symptom-based perspective has also allowed mental disorders to be defined as clinically significant symptoms reflecting dysfunction inside the individual (Wakefield, 1992), without taking into account the life events and social context in which the onset of symptoms takes place^2^.

In the case of depressive disorders, this new approach meant that there were no longer any grounds for the distinction between neurotic and psychotic depression, a distinction that relied on a psychoanalytic framework (Shorter, 2009, 2013); even more importantly, it was no longer possible to draw the more general distinction between endogenous and reactive depression, and therefore a wide and heterogeneous range of disorders that manifested with similar symptoms were placed under the same diagnostic category of MDD. For a diagnosis of MDD, an individual must present – on a close to daily basis – five of the following nine symptoms for a period of at least 2 weeks: (1) depressed mood; (2) anhedonia, that is to say, diminished interest in usually pleasant activities; (3) weight gain or loss or change in appetite; (4) insomnia or hypersomnia (excessive sleep); (5) psychomotor agitation or retardation; (6) fatigue or loss of energy; (7) feelings of worthlessness or excessive or inappropriate guilt; (8) diminished ability to think or concentrate or indecisiveness; (9) recurrent thoughts of death or suicidal ideation or suicide attempt. A diagnosis of MDD also requires the five symptoms to include either depressed mood or diminished interest or pleasure (American Psychiatric Association [APA], 1980, 2013a). According to Horwitz and Wakefield (2007), the problem with this kind of definition is that the mere presence of a particular group of symptoms is *sufficient* to diagnose the presence of a depressive disorder.

In *DSM-IV*, the grief experienced after the death of a loved one was considered distinct from other cases, because the same group of symptoms was viewed as representing a physiological reaction to a highly stressful negative event.

“Yet symptoms such as depressed mood, loss of interest in usual activities, insomnia, lessened appetite, inability to concentrate, and so on might naturally occur for a period of 2 weeks in the absence of any disorder after any of a wide range of negative events, such as betrayal by romantic partners, being passed over for an anticipated promotion, failing a major test that has serious implications for one’s career, discovering a life-threatening illness in oneself or in a loved one, or enduring the humiliation that follows revelations of disgraceful behavior. Such reactions, even when quite intense due to the severity of the experience, are surely part of normal human nature. Just as it is obvious why the *DSM* excludes bereavement from diagnosis, by parity of reasoning it seems obvious that it should also exclude these other sort of reactions to negative circumstances. The diagnosis, however, does not exclude such non-grief responses. Because of the symptom-based nature of the criteria, any sadness response involving enough of the specified symptoms for at least 2 weeks will be misclassified as a disorder, along with genuine psychiatric disturbances” (Horwitz and Wakefield, 2007, p. 9).

Thus, given that taking a person’s life events and social context into account in the diagnostic process may turn out to be too complex, modern *DSM*-informed psychiatry accepts the hypothesis that a diagnosis of major depression may be formulated on the basis of symptoms alone: in practice, the mere presence of five out of the nine depressive symptoms indicates that a particular individual, regardless of his/her life events and the meaning (s)he attributes to them, suffers from depression and as a consequence may be treated with antidepressant drugs. This approach is likely to result in the frequent mislabeling of normal sorrow (arising in response to negative events in a person’s life) as depressive disorder and, indeed, the last 20 years have seen an explosion in the US of flawed diagnoses of depressive disorders requiring pharmacological treatment, a pandemic largely driven by marketing pressures orchestrated by Big Pharma (Angell, 2004; Horwitz and Wakefield, 2007; Herzberg, 2009; Hirshbein, 2009; Greenberg, 2010). Although remaining “cautious about the possibility of incorporating context into diagnostic criteria and about the unreliability and false negatives that might result.” Spitzer (2007, pp. 8–9) himself has recognized this limitation of *DSM*: “… its criteria specified the symptoms that must be present to justify a given diagnosis but ignored any reference to the context in which they developed. In doing so, they allowed normal responses to stressors to be characterized as symptoms of disorder”. All these issues – he concluded – should be seriously considered as part of the agenda for *DSM-5*.

Despite the caveats coming from Spitzer, Frances, and many other eminent authors (see for instance Goldberg et al., 2010), the paradoxical decision finally made by the *DSM-5* task force was – instead of broadening the range of negative triggering events – to also expunge bereavement as a criterion for discriminating between normal and pathological depressive symptoms, relegating to a footnote the criteria for distinguishing depression from bereavement. This footnote, proposed by a European psychiatrist (Maj, 2014), specifies a number of clinical indicators such as a feeling of emptiness and loss alongside a preserved normal level of self-esteem in physiological grief versus anhedonia and a sense of self-disgust in depression, as well as recommending that clinical assessment take the patient’s overall personal history and cultural context into due account.

At this point, it is interesting to analyze the arguments used by the *DSM-5* task force to justify their omission of the bereavement exclusion clause (American Psychiatric Association [APA], 2013b).

The first argument concerns the fact that the period of 2 months following the loss of a loved one was arbitrary, given that “both physicians and grief counselors recognize that the duration is more commonly 1–2 years” (p. 5). The alleged “logical” consequence was that – in order to avoid any arbitrariness regarding duration – no time period should be indicated for normal grieving; thus, a person who has lost a loved one may potentially be diagnosed with clinical depression starting from the day of the funeral.

The second argument recognizes bereavement “as a severe psychosocial stressor that can precipitate a major depressive episode in a *vulnerable* individual” (p. 5, italics added). The concept of “vulnerability” is introduced as the basis for distinguishing between normal “physiological” sorrow over the loss of a loved one and a case of major depression. But, how is it possible to differentiate between these two kinds of suffering on the basis of symptomatic-behavioral criteria alone? Similar behaviors are observed in both cases, as the *DSM-5* editors seem to have acknowledged by dropping the bereavement exclusion clause. Even more importantly, “vulnerability” – like the opposite quality of “resilience” (Walsh, 2006) – is not an observable “fact”: it far more closely resembles a theoretical construct. But what kind of theory would underpin such a construct?

The third argument put forward helps to clarify this: “Bereavement-related major depression is most likely to occur in individuals with past personal and family histories of major depressive episodes. It is *genetically influenced*” (*ibidem*, italics added). The kind of vulnerability involved here seems to be “genetic,” and identifiable on the basis of “family histories” of major depressive episodes. Even if we leave aside the problematic nature of assessing in the “here and now,” on the strict basis of *DSM* symptomatic criteria, major depressive episodes in patient’s family members that may have taken place in the distant past, the only familial factors referred to here appear to be genetic ones: family histories of MDDs are merely considered in terms of their genetic influence on depressed individuals. However, family therapy and constructivist traditions might offer a very different view of shared family histories, for instance in terms of members taking part in the same family relational games (Selvini Palazzoli et al., 1989), family narratives (White and Epston, 1991), or family semantic constructs (Procter, 1996; Linares, 2010; Ugazio, 2013).

The fourth and final argument is highly significant for the line of reasoning that we go on to develop in this paper: “The depressive symptoms associated with bereavement-related depression *respond to the same psychosocial and medication treatments* as non-bereavement-related depression” (*ibidem*, italics added). The atheoretical and symptom-based nature of *DSM-5* allows different kinds of depression to be put together in the same category, *because they may be effectively treated using the same remedies, particularly medication*.

We would stress that while the first argument seems to be a matter of which convention to apply in relation to the duration of grief, the last three are based on an implicit assumption that is reductionist in nature, namely the assumption that concepts such as “vulnerability,” “familiarity” and the like may be reduced to their biological counterparts.

Thus, *DSM-V* reaches the conclusion that “*evidence* does not support the separation of loss of a loved one from other stressors in terms of its likelihood of precipitating a major depressive episode or the relative likelihood that the symptoms will remit spontaneously” (*ibidem*, italics added). What kind of “evidence” are we talking about here? In addressing this issue, let us sharpen our analysis of *DSM*’s atheoretical approach by focusing on two more general aspects.

The first aspect regards the difference between reliability and validity. *Reliability* refers to the degree to which a diagnostic system such as *DSM* allows two (or more) clinicians to independently agree on the diagnosis of a particular case, attributing it to the same category. *Validity* (or more appropriately *construct validity*) is the power of a given diagnostic category to actually represent and measure the phenomenon (i.e., the construct) it was designed for. Now, it is commonly held that *DSM has* addressed the need for reliability, which, as mentioned above, was a key unresolved issue prior to *DSM-III*, whose adoption meant that clinicians from different theoretical traditions could finally avail of a tool and a common “factual-symptomatic” language with which to compare and discuss their clinical assessments. On the contrary, however, *DSM* has been unable to offer robust construct validity for its own categorization of mental disorder. Such a flaw is not surprising if we consider the merely descriptive nature of the system, given that construct validity necessarily implies some kind of theory or model of the phenomenon to be studied that an atheoretical approach by definition cannot provide. Frances (2013) points out how difficult it is to find a balance between reliability and validity: the former imposes simplicity in order to make generalizations across all people suffering from a particular disorder; the latter tends to be subtle, complex, and inferential with a view to capturing clinical differences among individuals.

“If the criteria set includes items that are inferential or complicated, different clinicians will disagree on whether or not they are present. Worshipping at the temple of reliability, the *DSM* criteria sets are as simple as they can be – a catalog only of what is the most surface and common in mental disorders. This was a necessary choice, but it necessarily compromises validity-constraining ourselves to the simple blinds us to subtlety, nuance, and individual variability” (Frances, 2013, p. 23).

This is an inconsistency which is both theoretical and clinical: some of the main depressive symptoms are found not only in the case of bereavement or other negative events, but also in other forms of psychopathology (See Ugazio, 2013, p. 228).

“Thus the recent focus in psychiatry on reliability of diagnosis based on symptoms has been pursued at some cost to validity – that is, whether the diagnosis represents a correct attribution of disorder. The *DSM*’s criteria for MDD are one instance in which increased reliability has had the inadvertent side effect of creating substantial new validity problems” (Horwitz and Wakefield, 2007, p. 8).

The second aspect is, in a sense, more radical because it is of a philosophical nature: is it really possible to be atheoretical? One of the major conclusions from the philosophical analysis of science after the demise of logical empiricism in the second half of the 20th century has been that there is no such thing as notions of “experience,” “fact,” “evidence” and the like which are *not* theoretically informed. In the wake of the tradition begun by philosophers and historians of science such as Hanson and Kuhn, a commonplace of contemporary philosophy of science is that any piece of scientifically relevant ‘evidence’ is in fact *theory-laden* (Hanson, 1958), that is to say it is meaningful only when viewed as part of a theoretical framework – which Kuhn calls a *paradigm* – implicitly assumed by the scientist in order to make sense of phenomena (Kuhn, 1962/1970^2^). In the words of the philosopher of science Peter Godfrey-Smith,

“[…] a paradigm is a whole way of doing science, in some particular field. It is a package of claims about the world, methods for gathering and analyzing data, and habits of scientific thought and action. In Kuhn’s theory of science, the big changes in how scientists see the world – the ‘revolutions’ that science undergoes every now and then – occur when one paradigm replaces another. *Kuhn argued that observational data and logic alone cannot force scientists to move from one paradigm to another, because different paradigms often include within them different rules for treating data and assessing theories*” (Godfrey-Smith, 2003, p. 76).

Furthermore, mainly on the basis of Quine (1953) work in epistemology and the philosophy of language, contemporary philosophy of science questions the very possibility of drawing a sharp distinction in principle between purely “factual” statements, directly indicating “pure” experience, and “theoretical” statements (Quine, 1953: for a review of Quinean philosophy, see Hylton, 2014). In this sense, the “selectivity” of *DSM*’s atheoretical approach – which implicitly seems to draw a vague distinction between “abstract” and “factual” theories (whereby the latter resemble mere “facts” or “states of affairs”) – also appears to be ill-founded from a solely epistemological point of view.

## *DSM*’s NEUROBIOLOGICAL VIEW AND THE ISSUE OF “CIRCULARITY” IN THE DEFINITION OF DEPRESSIVE DISORDERS

It is widely recognized that the history of modern psychiatry has been profoundly influenced by the discovery and diffusion of drugs, particularly antidepressants (Kirsch, 2009; Shorter, 2009, 2013; Greenberg, 2010). In addition to the economic relationships between academic psychiatry and pharmaceutical producers, which many scholars and opinion leaders have condemned as one of the main reasons behind the contemporary depression pandemic affecting Western societies^3^ (Breggin and Breggin, 1994; Angell, 2004, 2011a,b; Cosgrove et al., 2006; Herzberg, 2009; Greenberg, 2010), the advent of psychopharmaceuticals has also played a crucial “epistemic” role in the theoretical definition of depression (Ehrenberg, 1998; Pignarre, 2001). The *criterion of efficacy* of the same medication in treating depressive symptoms regardless of context was one of the arguments on the basis of which the *DSM-5* editors decided to drop the bereavement exclusion clause. The effectiveness of a given type of drug (typically Selective Serotonin Reuptake Inhibitors, SSRI) on depressive symptoms is often taken as an established matter of fact, in terms of the observable effect of certain molecules on human behavior, and not as a theoretical hypothesis still awaiting full empirical support. The theory that a chemical imbalance in the brain causes depression seems to be widely accepted (Kirsch, 2009; Shorter, 2009, 2013). We will come back later to the weak points of such a theory, focusing for now on the *epistemic role* of the effectiveness of antidepressants, that is, the role played by drugs in defining the “validity” of the construct of “depression.”

First of all, it is important to note that, despite enthusiastic claims about advancements in neurobiological psychiatry, unfortunately no reliable biological markers have been found so far for the majority of mental illnesses, including depression. Nonetheless, recent neuroscientific discoveries about brain functioning obtained through neuro-imaging techniques (Legrenzi and Umiltà, 2012) are often taken as evidence justifying an almost exclusively neurobiological approach to the explanation of the mental realm. In describing the excessive ambitions associated with *DSM-5*, Frances (2013) writes:

“First was the unrealistic goal of transforming psychiatric diagnosis by somehow basing it on the exciting findings of neuroscience. This would be wonderful were it possible, but the effort failed for the obvious reason that it is still a bridge too far” (Frances, 2013, pp. 95–96).

Dowrick (2009), in reviewing the findings of the scientific literature on the biological basis of depression, adds:

“It would greatly assist the cause of those who see depression as discrete category or a disease entity if it could be demonstrated that it does have a unique and specific biological basis. Although psychiatrists such as Andreasen (2001) write as if such a basis has already been demonstrated, in reality this is far from the case. The search for a clear genetic explanation of depression – the holy grail of biomedical sciences and the pharmaceutical industry – has been extensive, arduous, and well-funded, but the results of this quest have not justified the enormous effort or expenditure” (p. 70).

This lack of evidence, though typical not only of psychiatry but also of many other branches of medicine (Maj, 2014), seems to be particularly significant for psychiatry. Together with the assumption that drugs effectively treat depressive symptoms on the one hand, and the *DSM* symptom-based criteria for depression on the other, it gives rise to a sort of “circular definition”: we label as *depression* the set of disorders whose symptoms are sensitive to the therapeutic action of antidepressants. In the absence of both a clear general definition of “mental disorders” (Wakefield, 1992; Frances, 2013; Maj, 2014) and of undisputed criteria for separating normal and abnormal depression, the drug efficacy criterion seems to have offered an easy way out. Pignarre (2001) describes such an approach as “small biology,” as opposed to “great biology,” which can provide solid proof of the causes of (organic) illness and has clear biologic markers at its disposal. Due to the fact that pharmaceuticals seem to act effectively on depressive symptoms although the causes of the disorder are still unknown, the drugs play a crucial role in the identification of the disorder itself. This definition is not represented by a set of necessary and sufficient conditions explaining the onset of depression, but by positive responses to drugs – in terms of symptomatic remission.

From such a perspective, the distinction between endogenous and reactive depression becomes useless: because antidepressants display similar efficacy in both cases, the traditional distinction may be abandoned, including in the case of bereavement. Psychopharmaceuticals, given their essentially symptomatic effects, are “practical” in nature, that is to say they are “atheoretical,” consistently with the *DSM* framework. In order to endow them with theoretical status, it is necessary to come down on the side of the “biological option” as fully explaining mental disorders:

“The major psychiatric illnesses are diseases … caused principally by biological factors, and most of these factors reside in the brain … As a scientific discipline, psychiatry seeks to identify the biological factors that cause the mental illness. This model assumes that each different type of illness has a different specific cause” (Andreasen, 1984, pp. 29–30).

Although this view still seems somewhat of a gamble, it has determined the success of medication-based treatment at the expense of other kinds of psychotherapeutic treatments such as family therapy, which search for contextual causes and remedies for (at least some kinds of) reactive depression (Pignarre, 2001).

Furthermore, it should be noted that *DSM* diagnostic categories were designed not only for clinical practice, but also for research purposes. The groups of homogeneous subjects suffering from a certain mental disorder who are eligible to take part in controlled studies within psychiatry are constructed on the basis of *DSM* categories. These subjects are often tested in terms of their reaction to different kind of treatments, especially drugs. But if, as we have argued, sensitivity to drug effects is part of the definition of the diagnostic categories, research designs too are likely to be compromised by a circular and self-confirming perspective.

Let us now turn to the controversial issue of drug effectiveness, from which the theory of chemical imbalance in depression derived most of its empirical support. It cannot be excluded that the effectiveness of treatment with medication might be due not only to the drug’s active principle, but also to other factors, such as spontaneous remission or a placebo effect. Kirsch (2009), whose initial research interest was the placebo mechanism, has seriously challenged what he calls the “myth” of the effectiveness of antidepressants. After conducting a meta-analysis of published studies conducted in this field, he also examined unpublished literature, requesting permission from the FDA (*Food and Drug Administration*) to access “secret” databases. Kirsch’s (2009) conclusions were astonishing: antidepressants work mainly on the bases of placebo effect. In particular, he found no significant difference between SSRI effects and placebos for light and moderate depression, while in the case of more serious depression the drug effect size was small. Moreover, psychotherapy seems to perform slightly better than drugs in terms of recovery (also at follow-up tests), while patients who received no treatment showed a significantly lower level of improvement than those who received any type of treatment (drug, psychotherapy, and placebo, respectively). In addition, when active placebos (i.e., inert substances with side effects) were used, there was no difference at all in the effects of antidepressants and placebos, a finding suggesting that the patients in double-blind randomized studies were able to guess which group they had been assigned to (drug vs. placebo) on the basis of whether or not they experienced side effects. Kirsch also compared studies using different drugs and found contradictory outcomes: depressive symptoms seem to be influenced in a similar way by medications that, respectively, increase and decrease serotonin levels; while drugs that have no impact at all on serotonin also seem to be effective^4^. These findings seriously question the widely accepted theory that antidepressants are effective, as well as the related theory that depression is explained by chemical imbalance in the brain. Kirsch draws a drastic conclusion in this regard: the account of depression as a chemical imbalance in the brain is simply wrong. Even if we do not wish to be so drastic, we are bound to conclude that there are many open questions in relation to the efficacy of antidepressants.

Studies conducted on non-human primates (McGuire et al., 1983; Raleigh et al., 1984; Sapolsky, 2005) suggest that serotonin levels vary as a function of primates’ social status. When dominant males were removed from their high-level hierarchical position in the group, they appeared to manifest “depressed” behaviors, refusing food, and engaging in a diminished level of activity; at the same time their serotonin levels rapidly decreased. On the contrary, those who replaced the dethroned males in superior positions displayed an enhanced level of activity, along with an increase in serotonin levels. Therefore, although depression in primates appears to be correlated to low levels of serotonin, the etiological theory of serotonin deficiency seems not to play the main role in explaining depression: “elevated blood serotonin concentration is a *state-dependent consequence* of active occupation of the dominant male social position, and we believe that a reinterpretation of the significance of hyperserotonemia in humans may be warranted” (Raleigh et al., 1984, p. 405).

## THE NORMATIVE NATURE OF PSYCHIATRIC DIAGNOSIS

Our analysis so far seriously questions the validity of both the epistemic value of the (a)theoretical *DSM* symptom-based definition of depressive disorders and the empirical validation of drug efficacy for their treatment. But even if, for the sake of argument, we were to leave these issues aside, another problem arises, namely the *normative* nature of both diagnoses and treatments in psychiatry (Stier, 2013). In general terms the status of normativity is an especially thorny issue, particularly against the contemporary background of naturalism (see De Caro and MacArthur, 2004; Laudisa, 2014 for a recent assessment of naturalism), currently by and large the prevailing approach to scientific knowledge. Assuming that our lives depend to a large extent on normative entities and issues, as well as on a biological, chemical, and physical structure, how do normative entities relate to the natural order? And do they *really* exist, or are they reducible after all to natural entities or processes? (See De Caro and MacArthur, 2010 for a recent assessment of normativity in relation to naturalism).

One of several instances of how normativity is relevant to our discussion concerns the notion of *normality*. Significantly Frances (2013) gave his book on *DSM-5* the title *Saving Normal*, devoting the entire first chapter to discussing “what’s normal and what’s not.” Now it is clear that this sort of analysis – far from being ‘factual’ – presupposes a reference to values and culturally negotiated standards, according to which a researcher formulates a true theory of “normal” and “(psycho)-pathological” phenomena that is highly normative in nature: in order to distinguish between normal and abnormal conditions, one cannot avoid assuming – at least tentatively – a notion of what “normality” means in a given social context, and no “fact” *per se* can provide such a notion.

“It would not be very shocking to claim that, e.g., neuroscientists have to use normative concepts such as the ‘correct functioning’ of certain brain areas. Nearly everything in the world – including psychiatry – is normative in this sense. A much more provocative claim is that psychiatry is guided by social, moral, cultural, and other norms. If this is true, and if it is also true that these kinds of norms are relative to time and place, then psychiatry cannot claim to know what a mental disease is ‘in itself’, where normality ends and mental disorder begins” (Stier, 2013, p. 2–3).

Something similar holds for the notion of *efficacy*, of which we can hardly make sense on the basis of merely factual or descriptive criteria. To say that a drug is “effective” means that it makes the patients feel *better*. If we wish to preserve their “heuristic” usefulness, at both the theoretical and practical clinical levels, we are forced to recognize that both the “normal–abnormal” continuum and the notion of “feeling better” are normative constructs that are not reducible to merely biological data. As Stier (2013) argues:

“whether something is a mental disease can only be determined on the mental level. This is so because we can only call behavior deviant by comparing it to non-deviant behavior, i.e., by using norms regarding *behavior*. Second, from this it follows that psychiatric disorders cannot be completely reduced to the physical level even if mental processes and states as such might be completely reducible to brain functions” (p. 1).

Moreover, with specific reference to depression for instance, what role is the concrete, lived experience of depression supposed to play in enriching the interpretive explanatory framework within which the pathology is located? It turns out to be plausible to claim that, in this regard, a crucial role is played by the patient’s own assessment of the relationship between self and the world: in “depressive” situations (Jacobs, 2013) patients fail to locate themselves in the world around them.

“Depressed persons often report that they feel disconnected from the world, that it appears as an empty place deprived of all meaning, that other people and activities formerly enjoyed are no longer of interest, that they get stuck in deliberative processes of rumination and indecisiveness, etc.” (Jacobs, 2013, p. 2).

If what is at stake here is making a *meaningful* connection with the world, it is far from surprising that such a highly evaluative and normative operation as that of attributing meaning to the relationship between oneself and the world eludes any biologically inspired, non-normative approach to psychopathology. However, any attempt to reduce the normative level to the empirical/biological level risks causing a significant loss in the analytical power of diagnostic categories (and consequently in the choice of clinical treatments). At a more general level, it means impoverishing the understanding, description, and explanation of mindedness.

## THE LOSS OF SUBJECTIVITY AND INTERPERSONAL CONTEXT

As stated above, *DSM* justifies its atheoretical perspective on the reasonable grounds that there is a need to pursue greater reliability of psychiatric diagnosis. In the case of depressive disorders this reliability has not proved to be so robust in either theoretical or clinical terms. Furthermore, validity issues seem to be unsolvable using an atheoretical approach. The problems in both of these areas appear to be connected to the cutting out of subjectivity and interpersonal context from the diagnostic process.

“A great deal is lost in the translation between the rich diversity of different individual experiences of depression and the bland five-of-nine criteria set chosen to define it. In describing the characteristics shared by those who meet the criteria for a given mental disorder, the *DSM* definitions must obscure the way they are individual and different. *DSM* definitions do not include personal and contextual factors, such as whether the depressive symptoms are an understandable response to a loss, a terrible life situation, psychological conflict, or personality factors” (Frances, 2013, p. 23).

The problem of subjectivity is well known to be one of the most thorny issues in the entire field of the sciences of mind. Not surprisingly the *DSM* symptom-based approach, inspired by evidence-based medicine (Pignarre, 2001; Shorter, 2009), attempts to objectify psychiatric mental disorders: in this view, subjectivity is a disturbance factor to be eliminated in order to purify scientific analysis of mental disorders. In particular, two kinds of subjectivity must be eliminated from *DSM*: (a) the subjectivity of the clinician making the diagnosis and (b) the subjectivity of the patient being diagnosed.

The former kind of subjectivity must quite obviously be sacrificed on the altar of reliability, by placing the clinician’s theoretical and etiologic beliefs on hold. In its extreme version, however, this approach is likely to reduce the diagnostic process to a mere checking off of symptoms against a list that is far removed from the person’s life as a whole. As Greenberg (2010) reports on the basis of his own experience, a psychiatric interview for diagnosing MDD may last about 7 min on average. Up to its fourth edition, *DSM* was formulated as a multi-axial system, made up of five dimensions on which the patient’s condition was to be assessed. In addition to the first axis assessing symptoms, which over time became the most important and often the only one actually used, it was possible to assess, via Axis IV, psychosocial stressors (e.g., death of a loved one, divorce, losing a job, etc.) that could affect the diagnosis, treatment, and prognosis of mental disorders; while Axis V was designed to evaluate the patient’s level of global functioning. This multi-axial approach has been omitted from *DSM-5*, in order to harmonize *DSM* with *ICD 10*, that is, the tenth edition of *International Classification of Disease* (World Health Organisation [WHO], 2010), a system that uses no axes. Moreover the axial system was considered too complex for practitioners to manage, with the risk that the diagnosis might be excessively biased by clinicians’ subjective judgment (Migone, 2013).

However, the most important omission from *DSM-5* concerns the subjectivity of the patient. The negative effects of excluding the relational context and life events of patients from the diagnostic process have been emphasized many times in this paper, taking bereavement as a paradigmatic example in relation to MDD. Likewise, our various citations of Frances (2013) and other authors illustrate how the validity problems of *DSM* are largely caused by its exclusive focus on symptoms, a perspective that overlooks the peculiar aspects of patients’ subjective experience. In addressing the question of normativity, we have argued that one of the major issues for depressive patients is having a meaningful relationship with the world (Jacobs, 2013). Now, we wish to stress here that the personal *meanings* individuals attribute to symptoms and their possible causes are as important as the symptoms themselves in diagnosing the kind of disorder patients are suffering from (Kelly, 1955; Neimeyer, 2009; Jacobs, 2013)^5^. Moreover, personal meanings are deeply embedded in the social, relational, familial context in which individual patients live: life narratives and discursive and cultural practices have a profound influence on many individual mental processes, both normal and abnormal (Bruner, 1990; White and Epston, 1991; Stolorow and Atwood, 1992; Neimeyer and Mahoney, 1995; Stern, 2005; Denborough, 2014). But exploring such meanings requires much more than a symptom-based checklist. It requires a *theory* enabling clinicians to establish “robust” connections between the different aspects of a patient’s experience. We are suggesting here that explanations in psychiatry and clinical psychology are at least in part based on *generalizations* of a particular kind, regarding both individual and relational processes in patients’ histories, the origin of psychopathology, and the onset of full-blown symptomatology (Guidano, 1987; Neimeyer and Mahoney, 1995; Neimeyer and Raskin, 2000; Arciero and Bondolfi, 2009; Neimeyer, 2009; Villegas, 2011; Ugazio, 2013). Such generalizations derive from a particular form of knowledge – acquired in clinical and psychotherapeutic settings – that concerns a relatively limited number of individual cases; although this kind of knowledge clearly bears less statistical weight than that obtainable in experimental settings, it has the advantage of being more in-depth and sophisticated in nature than the analysis of patients’ surface symptoms alone (Ugazio, 2013). The construction of this kind of generalization requires the adoption of an explicit theoretical standpoint enabling the formulation of hypotheses regarding subjective and contextual factors influencing the onset of symptoms.

The position just outlined is driven by a “double dissatisfaction.” Our first dissatisfaction is with psychological approaches based on generalizations defined in terms of overly “simple” operational constructs, which may only be evaluated via checklists of symptom or in controlled laboratory settings, that is, generalizations that do not take adequate account of the subjective and relational factors underlying the origin and manifestation of psychopathologies (Compas and Gotlib, 2002). But at the same time, we are also dissatisfied with positions denying the possibility that general statements may be formulated from clinical data, due to their idiosyncratic nature and the “complexity” of the subject matter (Anderson and Goolishian, 1992; von Glaserfeld, 1995): in the name of the uniqueness of each individual person and of the self-referential character of knowledge (particularly in relation to the human sciences), subjectivism and relativism risk confining clinicians within the narrow boundaries of the single case, preventing them from making, albeit tentatively, even the most necessary generalizations in terms of diagnosis and treatment (Ugazio, 2013).

## CONCLUSION

In this paper, through our analysis of the paradigmatic case of MMD and of the omission of the bereavement exclusion clause from *DSM-5*, we have argued that a solely symptom-based approach is seriously flawed, being based on an unduly restrictive view of mental disorders. Not surprisingly, such a view has justified a dramatic “medicalization” of normal psychological phenomena (such as mourning), causing increasingly widespread and indiscriminate use of drug treatments, and provoking strong reactions from some of the initial *DSM* supporters. From this strongly reductionist and naturalistic stance, mental disorders – despite their apparent peculiarity – are seen as essentially biological diseases, just like cancer or diabetes, and must be medically treated accordingly: in the near future technical tools and knowledge will be established that will allow us to reduce psychiatric symptoms to functional and/or chemical alterations in the brain (Andreasen, 2001; White et al., 2012). At the opposite end of the spectrum, advocates of anti-psychiatry (Szasz, 2001) maintain that mental disorders do not really exist *per se*, but rather are the outcome of the overwhelming pressure of cultural power on the weakest members of a given social system, a power that everyone is called to resist.

In more philosophical terms, if we appeal to the customary distinction (see for instance Moser, 2002) between ontology (concerning what there is in the world) and epistemology (concerning how we come to know what there is in the world), each of the above approaches tends to suppress one of these two aspects in exclusive favor of the other^6^. According to the reductionist and naturalistic approach, psychological and psycho-pathological phenomena are to be reduced in principle to their neurobiological correlates, because what exists in a fundamental sense is just the biological realm, whereas, according to the anti-psychiatric approach, mental disorders are to be reduced to culturally constructed artifacts. Thus both approaches appear to be ideologically driven and reductionist, given that they are both likely to prevent, in a theoretical and empirical sense, the fruitful integration of the neurobiological and sociocultural approaches within psychiatry and clinical psychology.

Are there any viable alternatives? The first could be the *Bio-Psycho-Social Model* (BPS), proposed by Engel (1977, 1980), that has been quite widespread as an alternative in psychiatry and in psychosomatic medicine to the reductionist biological model. BPS is supposed to be a model that combines both a philosophy of clinical care and a concrete treatment guide, with particular regard to the importance of the ‘patient-as-a-person’ in clinical relationship (Smith, 2002; Borrell-Carrió et al., 2004; Adler, 2009). From a philosophical perspective the BPS attempts to understand disease and illnesses by considering the multi-leveled organization of patients ranging from society to the biological. As a matter of fact, however, there is a wide debate about the validity and the consistency of this model.

On one side, it is considered as an important antidote to the reductionist biological psychiatry in as much as it reaffirms the importance of the psychological and social factors in understanding and treating mental disorders. Not by chance, BPS arose as an answer to the increase in the use of psychopharmacology linked to *DSM-III* (Ghaemi, 2009); so its caveats are still very relevant today (Adler, 2009, Helmchen, 2013).

But on the other side, it has been noted that “unfortunately, neither Engel himself nor his successors have ever provided clear-cut criteria as to how to use BPS characteristics to change the biomedical research paradigm” (Schubert, 2010, p. 389). According to these criticism, BPS never represented a real form of integration of the three levels, due to the fact that the interconnections between them remain unclear: such a general approach risks viewing the three levels (biological, psychological, social) as separate, given that their actual interrelations still require much additional analysis, both theoretical and empirical.

*Bio-Psycho-Social Model*’s more severe critics argue that it has just resulted in a form of vague eclecticism, not only due to the lack of concrete applications, but also due to the assumptions of the model itself (Ghaemi, 2009, p. 3). ‘The-more-is-better’ assumption of BPS does not necessarily provide a real advance, neither in the theoretical nor in the empirical realm: “An empirical defense of the ‘the more is better’ philosophy sometimes is made based on the eclectic biopsychosocial intuition that medications and psychotherapy are always, and inherently, more effective than either alone. Empirically, sometimes this is so, sometimes not. Using one method or treatment purely often produces better results or is more valid than using multiple approaches together” (Ghaemi, 2009, p. 4). From a philosophical perspective, BPS combines general system theory, psychoanalysis, semiotics, and constructivism (Adler, 2009; Schubert, 2010) in order to provide theoretical foundations to all of the three levels. Such a combination, according to the critics, does not prove to be fully consistent and robust. Stier (2014), in his commentary on Helmchen’s (2013) defense of BPS as an advanced, integrative, evidence-based conception of mental illness, argues that “the biopsychosocial model of mental illness is valuable as a reminder that there is more to mental illness than brain functions. Seen as theory, it will either be based on biology and meet similar trouble as the so called biologism in psychiatry, or else it will indeed be vague and border on anarchy” (p. 2).

Another more sophisticated alternative is represented by the so called “third wave of biological psychiatry” (TW). This framework supports what its proponents see as a growing caution in specifying the complex, multi-faceted nature of mental disorders, and argues in favor of a “multilevel approach ranging from genes to psychosocial mechanisms” (Walter, 2013).

First of all, although sensitive to the relevance of neurobiological correlation patterns, TW appears to be explicit on the amount of normativity implicit in any assessment of mental disorders: “According to the third wave of biological psychiatry, mental disorders are relatively stable prototypical, dysfunctional neural systems at various levels. *As with any understanding of disease in general the notion of a ‘dysfunction’ inevitably involves normative judgments of what is regarded as normal, functional, healthy on the one hand, and as abnormal, dysfunctional, pathological on the other hand*” (Walter, 2013, p. 2). Moreover, TW supports an approach to mental disorder in which sub-structures ranging from the biological to the socio-cultural level are seen as mutually interacting and linked by truly causal relationships. In order to provide a satisfactory explanation of mental disorders, a multilevel and multidimensional structure is postulated. All these levels are held to form what are called ‘mechanistic property clusters’ (MPC), with a terminology derived from a proposal originally presented by Kendler et al. (2011) and inspired by the application of the MPC concept to describe biological species (Boyd, 1991, 1999). In the latter case, morphological, physiological, and behavioral features appear to co-operate in order to characterize a species, although not all members need overlap in some single set of traits; “rather, members are clustered near one another in a feature space because of developmental, evolutionary, and physiological causal mechanisms and constraints” (Kendler et al., 2011, p. 1146). Kendler et al. (2011) support the extension to psychiatry of a similar approach: in this vein, we might conjecture the existence of a similar, complex and intertwined structure accounting for mental disorders, in which mechanisms ranging across the levels, albeit in a truly causal sense, can be conceived (although with a robust degree of idealization). Such kind of conjecture is also consistent with the claim that “etiological models for psychiatric disorders need to be pluralistic” (Kendler, 2008, p. 695; see also Kendler, 2005).

A final move of TW – a move that we prefer to remain neutral about – is to strengthen the link between psychiatry and philosophy of mind, on the basis of the claim according to which “if we better understand how mental states are related to brain states we might better understand how disordered mental states relate to disordered brain states” (Walter, 2013, p. 6). Our neutral stance on this option is motivated by what we see as a twofold risk. First, claims like the above seem to assume that mind–brain relation is a much easier issue than it really is: the ‘hardness’ of such an issue is often characterized as the circumstance that not only we do not know whether there is a solution to the mind–brain problem, but we are not even clear on what a ‘solution’ should look like. Second, even if we suppose that the mind–brain problem is not as hard as one might think, the sort of assumptions and style of reasoning typical of the philosophy of mind debates are likely to increase controversy that, far from positively contributing, might even make problems harder than they are.

Of course, like BPS, Walter’s TW is not exempt from criticism. Pawelzik (2013), although he recognizes the value of Walter’s proposal, points out some problems in the new wave of biological psychiatry. Firstly, being essentially “biological” and grounded in the methodology of neuroscience, it leads to a misguided vision of the mental realm, in which subjective consciousness is underestimated. Secondly, TW suffers from individualism: “mental functions –our ability to feel, to think, to act- are collectively defined, socio-cultural artifacts rather than purely natural, individual dispositions.” Therefore Pawelzik considers the “third wave as an individualistically limited enterprise” (p. 1). Thirdly, the brain plasticity, i.e., the possibility that experience – with particular regard to social experience deriving from *attachment relationships*- changes brain functioning is diminished: “If the mind that supervenes on brain states can actively change brain states, thereby redirecting the brain’s development depending on various environmental contingencies—than this ‘enactive mind’ is obviously underspecified by the third wave concepts Walter offers.” (…) “To sum up: Walter’s description of third wave biological psychiatry is on the right track: we should embrace his purgation of a lot of biologistic thought. Still (...), Walter left the main conceptual pillars of biological psychiatry—‘mindlessness’ and ‘medical model’—basically untouched (p. 2).”

If we turn back to *DSM,* it may well represent a useful tool among many others, and is therefore not to be considered (as is often the case) “the Bible of psychiatry” (Maj, 2014). It must be acknowledged that, in the section on “use of the manual,” the editors of *DSM-5* themselves warn about the risks of taking its symptomatic categories as the only criteria on which a diagnosis can be based. Symptom-based criteria may complement but cannot substitute reconstruction of patient’s clinical story and analysis of the social, psychological, and biological factors that may have influenced the onset of a disorder. In any case, checklists of symptom cannot convey the meaningfulness inherent in the clinical relationship with individual patients and the vivid knowledge to be obtained from “first-person” narratives of personal suffering (Frances, 2013; Jacobs, 2013). Furthermore, the provisional definition of mental disorders, formulated in the same section of *DSM-5*, specifies that pain caused by psychosocial stressors, especially the loss of a loved one, cannot be *per se* classified as a mental disorder: a claim that, as we have argued, can hardly be consistent with the omission of the bereavement exclusion clause and with the new definition of MDD^7^.

Apart from these criticisms, we acknowledge the useful role of *DSM* system: it provides some criteria as to whether and how people should be diagnosed and treated when they are unwell. We already stressed the prominent role played by *DSM* system in reaching reliability in the psychiatric field, a huge problem that today – from a general perspective – can be considered as resolved. Moreover, if it is true that *DSM* might invite a pathologization of normal experience, as a counterpart it might also be seen as promoting a “normalization” of pathological experience, thereby fighting some forms of stigma associated with mental problems.

In any case, our criticism is directed toward a specific disorder (MDD) in *DSM-5* and not toward *DSM* as a whole. The problem is not replacing the *DSM* system completely, but rather integrating it with other perspectives and tools (where applicable) not only on the “concrete” level of clinical practice (where the above integration is often adopted, e.g., in the so called “integrated approaches” that rely both on pharmaceutical treatment and psychotherapy), but also on a more general and theoretical level. It must be admitted that the overall consistency of such an integration in the current state of knowledge might result far from completely settled.

Moreover, a revision of the reductionist approach should consider the thorny problem of its feasibility. Ideally, one solution might be to create a multidisciplinary “ecumenical scientific manual,” in which all (or the major part of the) different perspectives and professional roles (i.e., psychiatrists, clinical psychologists, counselors, developmental/educational psychologists, family therapists, psychoanalysts, cognitive-behavioral therapists etc.) could be represented, discussed, and evaluated with regards to different mental disorders. Unfortunately, due to the presence of so many various points of view, there are limits to this kind of solution. The main ones are the risk of a reliability loss and the risk of high internal inconsistency: despite pointing at an integrated approach, such an attempt might result in a-systematic compilation of perspectives so distant that would be difficult to combine to create a worthwhile product^8^.

To some extent, it is a controversy that probably will never be resolved. Due to the fact that (at least in our opinion) the real problem is not to forcedly integrate opposing perspectives, but to clearly understand the assumptions entailed by a determined perspective and its domain of application -in Kelly’s (1955) words “the range of convenience” of a construct/theory-, perhaps it would be better if *DSM*, instead of declaring itself a-theoretical, could explicitly declare its theoretical position.

To sum up, we advocate what might be called a “moderate constructivist” meta-theoretical position. According to this position, it seems reasonable to view all diagnostic and clinical categories as provisional scientific constructs that can usefully guide both treatment and research, and to accept that the “normal–abnormal” continuum contains a structurally normative element (Pignarre, 2001; Frances, 2013; Stier, 2013). Thus, although it is possible to distinguish – in line with certain socially and culturally defined and theoretically informed scientific standards – the individuals that may be placed at each of the two extremes of the continuum, it turns out to be harder to position those falling in the middle.

As a concluding remark, we would like to stress that our argument here is meant to be founded not only on an enquiry into the empirical and theoretical structure of specific psychological and/or psychiatric theories, but above all also on a foundational and philosophical analysis reminiscent of a truly “humanistic approach” to the human sciences (Williams, 1991). It is our deep conviction that such an approach may contribute to paving the way for a more fruitful integration of neurobiological factors with cultural, social, familial, and personal meanings and values, toward achieving a richer and more valuable characterization of “human nature.”

## Conflict of Interest Statement

The authors declare that the research was conducted in the absence of any commercial or financial relationships that could be construed as a potential conflict of interest.

## Footnotes

1 *DSM* has acquired over time an increasing relevance, up to the current status of “Bible of psychiatry” (Maj, 2014). Given this status, its relevance has had a significant impact also on the work of other figures in the field of mental health, such as for instance clinical psychologists and counselors, who both in their clinical and research activity refer to *DSM* and to its diagnostic categories, and even for judges and lawyers, due to the wide and long-established use of *DSM* in criminology and forensic psychiatry.

2 It is interesting to recall that in the first edition of *DSM* (American Psychiatric Association [APA], 1952), mental disorders were defined in terms of *reactions* (schizophrenic, neurotic, depressive…), a definition which presupposed the existence of “external” factors causing mental suffering to the individuals (Greenberg, 2010).

3 It should be pointed out here that the US is one of the few countries in which advertising directly targeted at the consumer has been legalized and that “official” *DSM* diagnoses are required for insurance companies to refund the cost of treatment. Frances (2013), who was in charge of the *DSM-IV* task force, expresses a sort of *mea culpa* for failing to predict the pharmacological abuse that came about in this situation and for failing to formulate stricter criteria for the diagnosis of depression and other mental disorders (such as ADHD, social phobia, autism, and so on).

4 According to Kirsch (2009) such conclusions hold not only for serotonin, but also for the other neurotransmitters that are usually considered to be implicated in depression.

5 According to many psycho(patho)logical theories, in particular those informed by the constructivist paradigm, the problem of making sense of the self and the world is *the* problem of mental life (Bruner, 1991; Hermans, 2003). “A distinctive feature of constructivist perspectives in psychotherapy is a specific interest in processes of meaning construction (Neimeyer and Mahoney, 1995; Raskin and Bridges, 2002; Neimeyer, 2009). Guidano (1991, p. 56–60) defined psychopathology as a ‘science of meaning,’ developing the notion that ‘personal meaning organization’ guides the meaning making process underpinning the development of self, promoting coherence, and stability in personal identity” (Castiglioni et al., 2014, p. 120).

6 For a discussion of the distinction between ontology and epistemology within the constructivist paradigm to which we refer later, see Hacking (1999); Raskin (2001), and Castiglioni (2011).

7 For an alternative perspective on grief and bereavement, see Neimeyer et al. (2011).

8 Another thorny issue should be considered. As we mentioned in paragraph 3, the “circularity” of definitions of clinical and research levels with relation to depressive disorders must be avoided. With regards to the double role that DSM plays in clinical and research fields, we see two possible solutions: (1) DSM could play both roles, *if the different general assumptions underlying the two roles are clearly specified*; (2) the two roles could be separated, while at the same time integrated in order to provide reciprocal feed-back between clinical practice and research. But the solution is to be found case by case. It is not surprising that DSM manual is usually supported by clinical casebooks that present real “prototypical” life histories to exemplify the various mental disorders (see for instance Spitzer et al., 2006; Barnhill, 2014).
